# Utilitarianism

**Published:** 1854-09

**Authors:** 


*Utilitarianism.*—In a search among the contents of a portfolio, for matter suitable to fill a deficit in our editorial department of this month, we found the following, written some months since. It is not the habit of physicians to look upon their occupation æsthetically, or to feel any doubts as to its intrinsic dignity. All the toilsome and often disgusting details of medical study and life are looked upon as mere matters of scientific interest. So, too, in all branches of scientific pursuit; the physical overreaches the pschycical — the hard student in geology is known by his sunburned face — the chemist by soiled hands and garments — the pale face, the sunken eye growing dim by midnight watchings, are no longer the marks of a student. He has his work to do, often very toilsome; he sleeps of nights like any other laborer, wears a "wide awake" hat, and is a man among men.

In spite of this throwing aside the æsthetical dignities of the student, he has gained ground and acquired new importance in society. He has, in fact, become the recognized man of learning; to the great damage and indignation of the man of learning of fifty years ago, the recondite digger of Greek roots, the commentator on the Latin Poets, the admirer and impersonation of the Platonic philosophy.

This preface brings us to our MS.:

" It is, of course, a good thing to diffuse knowledge, to be the organ through which thoughtful minds may speak, to publish new discoveries in medicine, and so to lessen suffering and lengthen out the span of human life.

" But medicine finds only its end and perfection in those objects. Preliminary to their attainment, and going along inseperably linked with all true medical progress, is a broad array of natural science in its widest acceptation. No one of those studies which seek out the natural order of things, either in the animate or inanimate world, but has its interest to the student of medicine, and its bearing upon his pursuits.

" Hence it is that the journalist finds himself compelled to step aside from the history and treatment of disease, and search for those influences which, existing in the material world, are extended also to the phenomena of health and disease. It is in this field that he meets with difficult places.

" The pursuit of natural science, to which the medical profession is prone, has drawn upon it the censure and opposition of those who, clinging to the creeds and beliefs of an unscientific age, can only see in the new developments of scientific fact the downfall of their favorite doctrines.

" So strong has this feeling become that by its influence an alliance has been effected between the coarse prejudices of the uneducated man and the super-refinement of the metaphysical scholar. An instance of this was recently afforded in the opposition made to the anatomical law passed last winter. Fools and philosophers combined in that crusade. The coarse materialism of the ignorant was stayed up and sustained by the brilliant metaphysics of Platonic philosophy, and the solemn argument of a resurrection and a life to come. To that large number of our readers who are also readers of Harpers monthly, we refer the articles in the editor's table last winter from the pen of Prof. Tayler Lewis. It is not our intention to review these productions—that has already been effectually done by the American Medical Monthly. But we wish to call attention to the assertion in them made of the superiority of Platonic philosophy over scientific fact. In them the medical profession is derided as a coarse and materializing body of men, the patient gatherers of those isolated facts which together make up the sum of science are ridiculed as those who undertake an Herculean task with pigmy tools; and the division of labor among scientific men, the setting aside to different minds those special tasks to which their tastes and abitities lead them, draws down the scorn of this distinguished writer. He would have us, like the religionist and the metaphysician, seek for principles; for great truths which should rise above mere every day utility, and secure homage from their stately proportions. It is the old story of Grecian logic, the refusal to apply reason to the improvement of the arts as tending to degrade the human intellect."

(We omit sundry hard things here said in the original MS., as irrelevant to the issue.)

"It was undoubtedly very pleasant for Prof. Lewis to sit in his study and write down the spirit of the age. In the progress of science the mere man of Greek verbs and Horatian scholarship has come to be considered rather ornamental than useful. There is a stronger reverence for the steam engine than for statuary. We hope that classic attainment is not in its decadence; we would be very unwilling to see it excluded from the curriculum of our schools. It is a help to any man, it confers fluency and grace of language, it lies at the bottom of all logic, and all correct taste in morals or art. But it is not the moving spirit of the age, it does not sufficiently assimilate itself to utilitarian progress, and *therefore* it must assume a secondary place. It is finished and complete in its perfect proportions, nothing can be added to make it more so, and for that very reason it does not attract to it the inventive or the industrious mind. Studious men are thus directed to natural science as the only field in which anything may be originated; the great weight of the talent of the country has gone thitherward, and no cant concerning 'mere utititarianism' can restore the classic scholar to the same influence and eminence which have in all previous ages been his birth-right."

